# Polarised Multiangular Reflectance Measurements Using the Finnish Geodetic Institute Field Goniospectrometer

**DOI:** 10.3390/s90503891

**Published:** 2009-05-22

**Authors:** Juha Suomalainen, Teemu Hakala, Jouni Peltoniemi, Eetu Puttonen

**Affiliations:** Finnish Geodetic Institute, Box 15, 02431 Masala, Finland; E-Mails: teemu.hakala@fgi.fi (T.H.); jouni.peltoniemi@fgi.fi (J.P.); eetu.puttonen@fgi.fi (E.P.)

**Keywords:** Finnish Geodetic Institute Field Goniospectrometer, FIGIFIGO, reflectance, spectrum, BRF, HDRF, BRDF, spectrodirectional remote sensing

## Abstract

The design, operation, and properties of the *Finnish Geodetic Institute Field Goniospectrometer* (FIGIFIGO) are presented. FIGIFIGO is a portable instrument for the measurement of surface Bidirectional Reflectance Factor (BRF) for samples with diameters of 10 – 50 cm. A set of polarising optics enable the measurement of linearly polarised BRF over the full solar spectrum (350 – 2,500 nm). FIGIFIGO is designed mainly for field operation using sunlight, but operation in a laboratory environment is also possible. The acquired BRF have an accuracy of 1 – 5% depending on wavelength, sample properties, and measurement conditions. The angles are registered at accuracies better than 2°. During 2004 – 2008, FIGIFIGO has been used in the measurement of over 150 samples, all around northern Europe. The samples concentrate mostly on boreal forest understorey, snow, urban surfaces, and reflectance calibration surfaces.

## 1. Introduction

Reflectance, as a function of view and illumination directions, is described by the concept of Bidirectional Reflectance Factor (BRF). In short, BRF is defined as a ratio between radiance from the surface and a Lambertian white reference panel, while illumination and observation geometries are held constant [1]. Bidirectional means that the reflectance is treated as a function of both view and illumination direction.

Polarisation of light expresses the oscillation orientation of the electromagnetic field. Thermal light sources, such as the Sun and most lamps, produce unpolarised light, i.e. light that has an equally distributed mixture of different polarisations. However, when unpolarised light interacts with a surface, the scattering processes polarise the reflected light.

BRF is unique for each sample and varies over a large range; each sample has its own unique reflectance spectrum, polarisation behaviour, and directional distribution. This BRF behaviour depends on the geometrical and physical properties of the sample. For example, vegetation canopies with wax covered leaves tend to scatter light efficiently forwards, and canopies with 3D structure tend to have strong backscatter and generally enhanced reflectance on low view angles [2]. Thus it is clear, that multiangular observations have the potential to retrieve structural parameters of the sample.

Traditionally, remote sensing approaches have considered the view angle and solar zenith angle dependence of reflected radiation to be a problematic source of error, requiring a correction to a common geometry [3]. However, during the last decade, the remote sensing community has woken up to the possibilities of multiangular data interpretation; instruments such as MISR [4], POLDER [5], CHRIS/PROBA [6], and various airborne sensors are able to collect multidirectional radiances and reflectances. Knyazikhin *et al.* [7], García-Haro *et al.* [8], and Gascon *et al.* [9] have applied these data to retrieve forest leaf area indexes. Sandmeier *et al.* [10] applied BRF for boreal forest classification and structure analysis. Bourgeois *et al.* [11] stated that an accurate determination of surface albedo requires multiangular observations.

Similarly, the land remote sensing community has commonly ignored the polarisation of reflected light. Most of the operational sensors are insensitive to polarisation of light, but e.g. POLDER satellite sensor, its airborne version OSIRIS [12], and the to-be-launched Aerosol Polarimetry Sensor (APS) satellite [13], are capable of taking polarised measurements. The polarisation capabilities of these two satellites are mainly designed for atmospheric measurements, but they can also be used for the remote sensing of ground cover.

BRF measurements, with and without polarisation, are needed for various applications: First, basic knowledge on BRF and polarisation effects are needed to assist in the development of optimal sensors and algorithms. Second, empirical BRFs are needed as input parameters for various reflectance models. Third, all satellite and airborne reflectance data products and reflectance models need to be validated. Often, the easiest way to do any of the above mentioned is by using empirical BRFs.

BRF measurements are usually taken using goniospectrometers, often referred only as goniometers. A goniospectrometer is a system consisting of a spectroradiometer and mechanics that change its view direction. One of the first well-known instruments was at the European Goniometer Facility (EGO) [14]. EGO is a laboratory system that rotates the spectrometer in a hemisphere around the sample at approximately a 2-meter radius, and this way measures the reflected light from selected view angles. To gain a good representation of the BRF, the measurements are repeated at multiple illumination angles.

Measuring reflectance factors in a laboratory has many advantages. First, the illumination geometry can be controlled freely. Second, weather and atmosphere effects don't affect the results. Third, in a laboratory, there are usually no critical time limitations for the measurement. Thus, in a laboratory, the samples can often be prepared and documented better than in the field. However, in some cases, *in-situ* measurements are required, e.g. if measurements are required to be simultaneous to aerial data or if the samples cannot be moved to a laboratory. Also, differences in laboratory illumination and sunlight produce challenges in direct comparison of field data and laboratory results.

The optimal properties of a field and a laboratory goniospectrometer are quite different from each other. For a laboratory-only instrument, a sturdy structure with a high level of automation is usually advantageous, because this provides the best pointing accuracy and repeatability. On the other hand, for field operation, the goniospectrometer should be portable. Speed of operation is also a critical property, as the prolonged measurements are often disturbed by movement of the Sun or the passing of clouds.

To address the need for *in-situ* measurements, special field goniospectrometers have been built. The majority of the large-scale field models are based on the design of EGO [2,15,16]. Although, these instruments have been adapted to field operation, they are heavy for manual transportation. They require either special transportation equipment or laborious assembly at the measurement location. If the instrument cannot be taken onto a rough ground or far from roads, the types of possible samples are limited.

Some smaller field goniometers [17,18] have also been built, but usually their design has compromised the measurement distance. The distance affects how large the sample can be, while the solid angle of observation is held reasonable. Having a large sample is advantageous when heterogeneous samples are measured, because this improves the sample representativeness. Sample heterogeneity is a significant problem especially when measuring natural vegetation samples.

This article describes the design, operation, and properties of Finnish Geodetic Institute Field Goniospectrometer (FIGIFIGO). The motivations of main design features are discussed, in order to promote the design of future field goniospectrometers. Section 2 describes the algorithms used in reflectance factor retrieval. Sections 3 and 4 present the design and operation of the instrument. Section 5 discusses the error sources in reflectance factor retrieval. Section 6 presents some example data from our measurements.

## 2. Methodology

### 2.1. Measurement of reflectance factor

**Figure 1.**
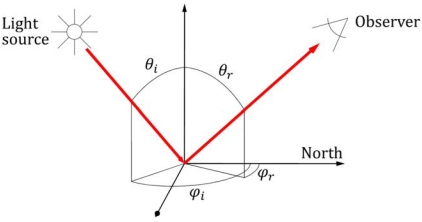
Bidirectional reflectance geometry. θ_i_, θ_r_, φ_i_, and φ_r_ are respectively the zenith and azimuth angles of incident (i) and reflected (r) radiance.

*Reflectance Factor* (*R*) is defined as [1]:

(1) $$R_{S}=\frac{L_{S}\left(\theta_{i},\varphi_{i},\theta_{r},\varphi_{r}\right)}{L_{id}\left(\theta_{i},\varphi_{i}\right)}$$

where L_S_ and L_id_ are the reflected radiances from a sample and an ideal Lambertian standard surface measured in the same illumination conditions. θ_i_, φ_i_, θ_r_, and φ_r_ are the zenith and azimuth angles of incident and reflected radiance as defined in Figure 1.

In practice, reflectances are always calculated against a non-ideal reference panel. If a reference panel with high isotropy is used (e.g. Spectralon 99%), the angular dependency of R_ref_ can be omitted. If absorptions in the panel are taken into account, we can write:

(2) $$R_{S}=\frac{L_{S}\left(\theta_{i},\varphi_{i},\theta_{r},\varphi_{r}\right)}{L_{\text{ref}}\left(\theta_{i},\varphi_{i}\right)}R_{\text{ref}}$$

This is a general equation for reflectance factor acquisition using a reference panel with isotropic reflectance.

In the case where both illumination and observation are unidirectional, e.g. in laboratory measurements, Equation 2 produces *Bidirectional Reflectance Factor* (BRF). *Bidirectional Reflectance Distribution Function* (BRDF) is closely related to BRF, and an approximation for it can be produced simply by dividing BRF by pi. Similarly, Equation 2 returns a *Hemispherical Directional Reflectance Factor* (HDRF) if the distribution of illumination is hemispherical and observation direction is directional. [1] This is the case in typical sunlight measurements, where there is a diffuse (blue sky) component present. To acquire BRF in natural sunlight, the reflected radiance is split into two components:

(3) $$L\left(\theta_{i},\varphi_{i},\theta_{r},\varphi_{r}\right)=L^{\text{dir}}\left(\theta_{i},\varphi_{i},\theta_{r},\varphi_{r}\right)+L^{\text{diff}}\left(\theta_{r},\varphi_{r}\right)$$

where *L* is the total reflected radiance, and *L^dir^* and *L^diff^* are its components originating from direct and diffuse illumination. Total reflected radiance (*L*) measured from a surface is the output of a default spectroradiometer measurement. If only the direct component of the incident radiation is blocked (i.e. the sample is shadowed) the similar measurement yields the diffuse component (*L^diff^*). Thus BRF can be acquired, even in natural sunlight illumination, by exploiting both total and diffuse radiance measurements:

(4) $$R_{S}^{\text{BRF}}=\frac{L_{S}\left(\theta_{i},\varphi_{i},\theta_{r},\varphi_{r}\right)-L_{S}^{\text{diff}}\left(\theta_{i},\varphi_{i},\theta_{r},\varphi_{r}\right)}{L_{\text{ref}}\left(\theta_{i},\varphi_{i}\right)-L_{\text{ref}}^{\text{diff}}\left(\theta_{i},\varphi_{i}\right)}R_{\text{ref}}$$

In typical measurement conditions, the effect of diffuse illumination is concentrated strongly on UV and blue light. On longer wavelengths, the effect of correction is typically only of a few percent of the signal. While a natural variation of the samples already produces errors much larger than this, it is well justified to approximate reflected diffuse radiances to stay constant over view direction. This approximation quickens the measurement process significantly by enabling the use of one nadir diffuse measurement for all sample radiances.

### 2.2. Variations in incident irradiance

Unfortunately, it is often impossible to measure all radiances in eq. 4 simultaneously, which leads to errors if there are variations in the incident irradiance. The error caused by this can be reduced if an independent instrument is used to record incident irradiance in real time during measurements. Incident irradiance (*E(t)*) can be used to compensate for illumination changes by substituting all radiances with instant reflectances (*ρ*):

(5) $$L\to \frac{L\left(t\right)}{E\left(t\right)}=\rho (t)$$

A similar correction has also been suggested by Schopfer *et al.* [15]. By implementing the previous changes, we get the equations for HDRF and BRF acquisition in sunlight:

(6) $$R_{S}^{\text{HDRF},\text{sunlight}}=\frac{\rho_{s}(t_{1})}{\left[\rho_{\text{ref}}\right](t_{1})}R_{\text{ref}}$$

(7) $$R_{S}^{\text{BRF},\text{sunlight}}=\frac{\frac{\rho_{s}(t_{1})}{\left[\rho_{\text{ref}}\right](t_{1})}-\left[\frac{\rho_{s}^{\text{diff}}(t_{2})}{\left[\rho_{\text{ref}}\right](t_{2})}\right](t_{1})}{1-\left[\frac{\rho_{\text{ref}}^{\text{diff}}(t_{3})}{\left[\rho_{\text{ref}}\right](t_{3})}\right](t_{1})}R_{\text{ref}}$$

where *t_1_, t_2_*, and *t_3_* are respectively the measurement times of sample, diffuse sample, and diffuse reference panel radiances, and notation *[] (t)* stands for linear interpolation of bracket contents to time *t*. These interpolations are done in order to minimize the effects of time-dependent illumination changes.

### 2.3. Polarised reflectance factors

If the radiometer is equipped with a linear polariser and the above-mentioned measurements are taken with multiple polariser orientations, polarised reflectance factors (*R_S_P_*) can be calculated. To calculate these, the following substitutions need to be made in equations 2, 6, and 7:

(8) $$R_{S}^{*}\to R_{S\_P}^{*}$$

(9) $$\rho_{S}^{*}\to \rho_{S\_P}^{*}$$

(10) $$\rho_{\text{ref}}^{*}\to \left(\rho_{\text{ref}\_P}^{*}+\rho_{\text{ref}\_P+90{}^{\circ}}^{*}\right)$$

where *P* is the orientation of linear polariser; *P+90°* is an orientation perpendicular to orientation *P*; and * states that substitution is done to both diffuse and total radiance variables.

## 3. Instrument Description

**Figure 2.**
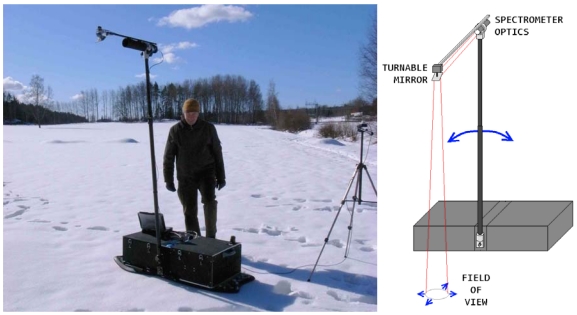
(Left) FIGIFIGO measuring snow at Kirkkonummi, Finland. (Right) Diagram of FIGIFIGO with directions of automated movement (blue arrows). The view zenith angle changes as the arm turns from side to side. The position of the footprint is stabilized with small mirror adjustments. When needed, two laser pointers (red lines) highlight the footprint position.

The Finnish Geodetic Institute Field Goniospectrometer (FIGIFIGO) is a portable instrument for bidirectional reflectance factor measurements (Figure 2). Optionally, polarising FIGIFIGO optics can be used for the measurement of linearly polarised reflectance factors. The basic FIGIFIGO configuration consists of a goniometer body, a turning arm, and a laptop computer. Accessories include a sunphotometer (SP-Lite, Kipp&Zonen, Delft, The Netherlands) on a tripod for field configuration, and a rotation base and laboratory illumination system for laboratory configuration.

### 3.1. Goniometer body and electronics

The goniometer body is a box (103 × 51 × 27 cm) containing the spectrometer and most of the system electronics. All electronics are powered by a 12 V lead acid battery. The electronics include a brushless DC motor (Series 4,490,048 BS with 352/1 gearbox, Faulhaber, Schönaich, Germany) to turn the arm, a 12-bit analog-to-digital converter (LabJack U3, LabJack Corporation, CO, USA) for sensor integration, and various supporting electronics. All goniometer actions are controlled with a rugged laptop computer (Panasonic ToughBook CF-18) over a single USB cable. The control software with a touch screen interface is built on National Instruments LabVIEW development environment.

The main sensor of FIGIFIGO is a FieldSpec Pro FR (Analytical Spectral Devices, Boulder, CO, USA) spectroradiometer (350 – 2,500 nm) that is compartmented inside the goniometer body. A 3-meter optical fibre of FieldSpec runs to the optics at the end of the arm. The spectrometer is connected to the computer using an ASD Smart Ethernet adapter.

For determination of Sun position and goniometer heading, the system has a consumer grade GPS receiver, a hemispherical sky camera (uEye UI-1645LE-C-HQ, IDS, Obersulm, Germany), and a goniometer body inclinometer (SCA121T-D07, VTI, Vantaa, Finland). The Sun's position is calculated using GPS coordinates and time. The goniometer azimuth heading is then determined with an algorithm exploiting the absolute Sun position, a relative Sun position from sky camera image, and the tilt of the goniometer body. Until summer 2008, an electronic compass (C100, KVH Industries, Middletown, RI, USA) was used for azimuth determination, but its operation was found to be unreliable in urban areas due to steel reinforcements and structures. The compass is still exploited e.g. in night time field measurements in lamp light.

### 3.2. The arm and the optics

The arm is mounted directly to the axle of the Faulhaber motor. The root of the arm contains a two-axis inclinometer (SCA121T-D03, VTI, Vantaa, Finland) that determines the angle of the arm even if the body is not level. The arm length can be adjusted, with a telescopic mechanism, between 155 and 265 cm, depending on the desired footprint size.

**Figure 3.**
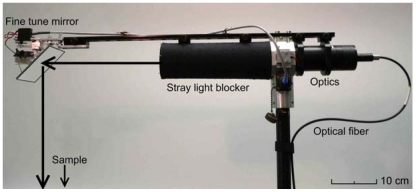
Optical system of FIGIFIGO. The spectrometer optical fibre is connected to the changeable optics. The optics point to the sample through a fine tune mirror. The mirror system allows the computer to fix the optics footprint to selected target without parallax error.

The top of the arm (Figure 3) holds the goniospectrometer optical system. The system consists of changeable optics, a fine tune mirror system, and two laser pointers for optics footprint determination. The optics point to the sample with a custom fine tune mirror system. The system allows the computer to continuously adjust the location of the spectrometer footprint. This is necessary in order to compensate for parallax, occurring if the sample is not positioned exactly on the arm axis.

With changeable optics, FIGIFIGO can be adjusted for measurements of different types of samples. In addition to the original ASD spectrometer optics, two sets of custom optics have been built. Both sets have footprints of 10–15 cm, depending on the used arm length. The polarising optics (*FGI_3deg_Polarising_2008a*) were built by mounting a broadband Glan-Thomson linear polariser in a computer-driven rotator (NSR1, Newport Corporation, Irvine, CA, USA). Tests with polarising optics showed that both SWIR sensors of our spectrometer were sensitive to polarisation. Even slight movement of spectrometer optical fibre caused changes of up to 20% in the received signal in the SWIR sensors. A similar effect was not experienced when measuring unpolarised light. To counter this polarisation sensitivity, it was necessary to also add a broadband wedge depolariser between the polariser and the spectrometer fibre. Because many samples reflect polarised light, it was also found necessary to replace the original ASD optics with a set of depolarising optics (*FGI_3deg_2008a*).

### 3.3. Laboratory equipment

In laboratory operations, the weight of the system is not limited by the requirement of portability. Thus in the laboratory, FIGIFIGO is coupled with a rotating steel base (2-meter diameter) for azimuth rotation. The base has a built-in encoder for automated determination of the goniometer azimuth angle.

**Figure 4.**
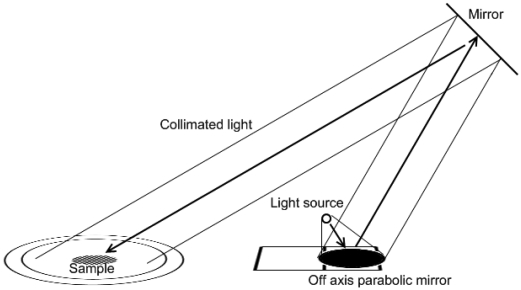
Illumination system. A 1,000-Watt QTH light bulb is positioned in the focal point of an off-axis paraboloid mirror (Ø 53 cm, f 47 cm). The collimated beam is pointed to the sample using a flat mirror. The illumination zenith angle can be varied freely between 20° and 70° by moving the flat mirror.

When sunlight is not available, a 1,000-Watt QTH light source (Oriel 66886 and 69935, Newport Corporation, Irvine, CA, USA) is used for illumination. To mimic sunlight in measurements, a collimated beam 30 – 70 cm in diameter is required. Both the original Oriel lens optics and later our own enhanced lens optics had significant problems producing an ideal beam: The beam was rather more conical than well-collimated; the light source filament shape was reproduced as brighter areas; and on the edges of the beam, it was possible to see a rainbow effect due to chromatic aberrations of the lenses. To improve the beam, the originally transparent light bulb was sanded to matte and the lens optics were replaced with an off-axis paraboloid mirror (Ø 53 cm, f 47 cm, off-axis angle 45°) carved from an aluminium block. The improved illumination system (Figure 4) consists of the light source, the parabolic mirror, and a flat mirror on a tripod. While the light source and paraboloid mirror remain on the floor level, the illumination zenith angle is varied by lifting and tilting the flat mirror.

## 4. Measurements and Processing of Data

Portability has been one of the main interests in the FIGIFIGO design. During transportation, FIGIFIGO is separated into smaller parts for easy and safe handling. With the arm removed, FIGIFIGO can be fitted in any estate car with space remaining for three or more passengers. In its field configuration it weighs only 30 kg with batteries and can thus be carried to even a relatively distant measurement site.

At the measurement site, the first thing is to attach the arm to the goniometer body and install the optical system to the arm. The set-up usually takes 5 – 15 minutes before the first sample, but transition to another nearby sample can be done in just a few minutes.

Next, reference measurements are taken. A white Spectralon reference panel is positioned in the optics field of view and carefully levelled using a bubble level. A white reference spectrum is recorded from nadir view direction. To enable BRF acquisition in sunlight, with equations 6 and 7, the reflected diffuse radiance from the Spectralon panel is measured from nadir by shadowing the panel with a 50-cm plate from a distance of 2 – 3 meters. Diffuse radiance from the sample is measured similarly.

As a standard procedure, these white reference and diffuse measurements are repeated at the end of the measurements. They are also repeated in between azimuth turns, if found necessary, e.g., if clouds are present or the measurements are long-lasting. If the polarising optics set is used, all of these calibration measurements are taken with each polariser orientation.

After the system is set up and the reference spectra are collected, the sample is measured by repeating the following steps: (1) The goniometer is rotated to a desired azimuth heading, and positioned so that the sample is in the optics field of view. (2) A measurement sequence is driven. During the sequence, FIGIFIGO registers the system position and the incident irradiance in real time; while the arm drives slowly (5 – 10 °/s) from the selected zenith angle (typically 60 – 80°) to the opposite side, continuously recording radiances. Point density in zenith direction can be controlled by varying the arm speed. If the polarised optics are used, the sequence consists of one zenith turn for each polariser orientation.

**Figure 5.**
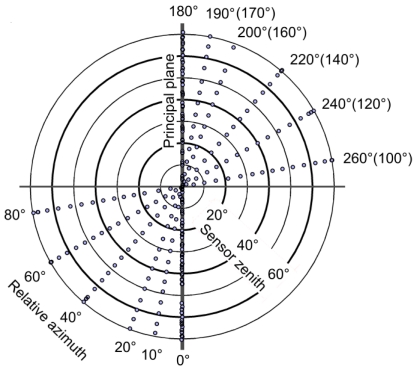
Measurement angles in a typical FIGIFIGO dataset. The dots depict sensor zenith (θ_r_) and relative azimuth (φ_i_ – φ_r_) angles of the measured spectra. If left-right symmetry can be assumed for the sample, the measurement typically consists of seven zenith turn sequences. The most distinct characteristics are usually seen at the principal plane. Thus near the principal plane, the point density is increased by measuring the 0°-sequence twice and measuring the additional sequence at 10°. The density of the points in zenith direction can be varied by changing the rotation speed of the arm.

When left-right BRF symmetry can be assumed for the sample, measurements at six azimuth directions (Figure 5) are usually found to adequately characterise BRF at one illumination zenith angle. The measurement pattern is denser near the principal plane, because most of the characteristic BRF effects are usually seen there. Additionally, in order to verify characteristics, to still increase the point density, and to take changes in sun elevation into account, the principal plane is measured twice, as the first and the last sequence. Such hemispherical measurements take typically 15 – 20 minutes. Polarised measurements take approximately 1.5 – 4 times longer, depending on the number of polariser orientations and spectrum sampling density.

After the measurements are finished, the raw data is taken back to the office and post processed. The radiances and metadata are checked for possible errors, and the documentation is completed. The radiances are processed to reflectances using algorithm, applying equations 2 and 6 – 10. The checked and processed data files are stored to a reflectance library.

## 5. Error Analysis

The errors in BRF acquisition can be divided into roughly three categories: First, radiometrical errors from the spectrometer measurement, white reference calibration, and variation of incident light; Second, angular accuracy from both angle registration and optics opening angle; Third, representativeness of the sample. Each of these categories is more accurately discussed in the following sections.

### 5.1. Radiometrical errors

**Figure 6.**
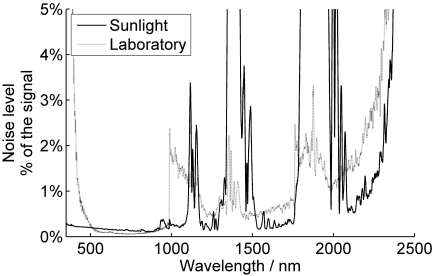
Typical noise levels in FIGIFIGO measurement of a white Spectralon panel. With darker samples, the noise levels are respectively higher. Sunlight measurement was made on an August afternoon in southern Finland (Sun zenith 54°). In the laboratory, the illumination zenith angle was approximately 45°. The sunlight spectrum shows increased noise at the atmospheric absorption bands around 1,200 nm, 1,400 nm, and 1,900. In the laboratory, the lower light levels enhance the noise at the spectrometer VNIR, SWIR1, and SWIR2 sensor borders (986 nm and 1,760 nm) as the sensitivities drops.

The error sources in FIGIFIGO radiometry follow generally the same principles as any spectrometer measurements. Noise in reflectance spectrum varies over wavelengths depending on sensor sensitivity and intensity of light. (Figure 6) In sunlight measurements, the noise is increased at the atmospheric absorption bands; around 1,200 nm, 1,400 nm, and 1,900 nm. In laboratory measurements, these absorption bands produce a decent signal, but otherwise the noise levels are the same or higher than in sunlight. Due to low irradiance of laboratory illumination, the spectrometer needs to use high gain at the SWIR sensors. Thus, also, noise at the borders of the SWIR sensor (986 nm and 1,760 nm) is amplified.

All reflectance factor measurements are produced as a ratio between radiances from the sample and the reference panel. Thus any variation in reflected radiance from the panel causes a systematic error in reflectance; e.g. the cleanness of the Spectralon panel produces an uncertainty of ±1% to all reflectances. Also, if the reference panel or the sample is tilted in the plane of illumination, its brightness changes according to the following equation:

(11) $$\frac{E_{\Delta \theta }}{E_{\text{ideal}}}=\frac{\cos\left(\theta_{i}-\Delta \theta \right)}{\cos\left(\theta_{i}\right)}\approx 1-\Delta \theta \tan\left(\theta_{i}\right)$$

where *Δθ* is the tilt of the target surface towards illumination (in radians); *θ_i_* is the illumination zenith angle; and *E_ideal_* and *E_Δθ_* are the incident irradiances received by a perfectly level and a tilted surface. The effect is enhanced at low illumination angles. In FIGIFIGO measurements, the reference panel is always balanced using a bubble level, with accuracy of ±1°. Thus e.g., at solar zenith angle of 60°, this causes an uncertainty of ±3% in all reflectances.

During the reflectance factor acquisition, atmospheric effects in incident irradiance are compensated using equations 5 –7 exploiting readings from the sunphotometer. Ideally, a second spectrometer should be utilized, but in the current FIGIFIGO set-up only a single band sensor is used. The silicon based sunphotometer responses only to radiation between 400 – 1,100 nm, and is unable to represent any spectral effects. However, usage of even monochrome irradiance data is bound to produce better results than not using any correction. Either way, the recorded irradiance time series provides an essential tool for detection of cloud interference.

### 5.2. Angular accuracy

Both sets of FIGIFIGO optics collect light with approximately 3° opening angle. Thus, strictly speaking, FIGIFIGO does not measure in bidirectional geometry as defined for BRF, but in directional-conical (or even biconical) geometry. [1,19] In order to achieve faster operation, FIGIFIGO collects spectra while the measurement arm is in movement. Also, due to finite spectrometer integration time, the measurement is not collected from a single direction but from a short range of zenith angles up to 1° long. However, the difference between a conical and directional reflectance factor can be seen only if there are significant second derivative angular effects at the scale of the optics opening angle. In practice, natural samples tend to have quite smooth BRFs with no radical features outside direct backscatter. Thus conical geometry of most FIGIFIGO measurements can be safely ignored.

Due to the arm movement, the inaccuracy of spectrometer timings, and mechanical factors, the uncertainty in sensor zenith angles registered with spectra is ±2°. The sensor azimuth angle accuracy of the preceding compass system depended heavily on the environment. Even in an environment without clear magnetic disturbances the actual azimuth accuracy was only ±5°. The current configurations, with solar camera system (added in the summer of 2008) and laboratory base encoder system, can both reach azimuth accuracy better than ±1°. The solar position is calculated from GPS time and coordinates with accuracy better that ±0.1°. In the laboratory, the illumination direction is defined with accuracy better than ±1°.

### 5.3. Spectrometer footprint variation and representativeness of the sampling

The spectrometer footprint varies slightly between data points due to the following reasons: (1) The footprint elongates as 1 / cos(θ_o_) with the view zenith angle. (2) If the sample is not exactly on the level of the arm axis, the footprint moves with the view zenith angle. Since autumn 2007, FIGIFIGO has had the fine tune mirror system that fixes the footprint location within ±1 cm. Earlier, the location was changed by the parallax error, if the sample was at different height than the goniometer axle. However, if the sample has a 3D structure, some view area variation is always unavoidable. (3) During the azimuth rotation around the sample, pointing errors of a few centimetres may occur, if positioning is not done with special care. (4) The optics footprint size varies slightly also spectrally due to the chromatic aberration of lenses. A variation of 2 – 4 cm in footprint diameter is experienced with the FIGIFIGO optics over the range of 350 – 2,500 nm. (5) The three sensors of the FieldSpec spectrometer use different fibres in the optical fibre bundle. Thus, while the total footprint shape is spherical, each of the three sensors does see slightly different parts of the sample. With heterogenic samples, this effect often produces clear “steps” in the spectrum at the sensor switch wavelengths.

The actual error in HDRF/BRF retrieval caused by the footprint variations is impossible to define explicitly. In sunlight, the impact depends solely on the heterogeneity of each target. With artificial illumination, also inhomogeneity of the light beam affects the error. In the end, the question of the footprint variation falls back to the representativeness of the sampling. Biasing of the sample can easily be the largest error source of the whole process, e.g. if the BRF retrieved from one 20-cm spot is assumed to represent a larger area or whole sample species. It is common to see variations of tens of percents within samples of the nominally same species, measured on the same day, at the same approximate location. The changes in moisture, soil, annual and diurnal cycle, dustiness, etc., still add their effects. Thus no technical improvement can annul the need of averaging over wide sampling.

## 6. Data

**Figure 7.**
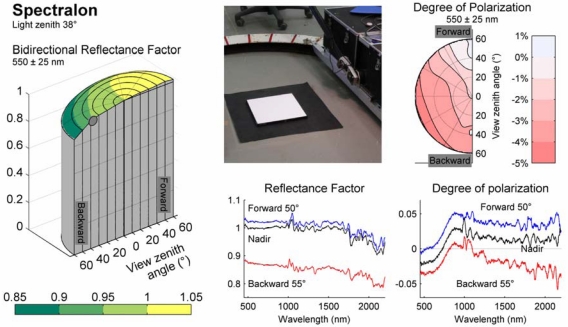
Reflectance properties of white Spectralon panel at 38° illumination zenith angle. (Top middle) Photograph of the target (Left) Bidirectional reflectance factor of Spectralon at a green spectral band as a function of view direction. (Bottom middle) Reflectance factor spectrum at three view angles on the principal plane. (Top right) Degree of polarisation ({*H* - *V}/{H* + *V}*) of reflected light at a green spectral band as a function of view direction. (Bottom right) Degree of polarisation spectrum at three view angles on principal plane

**Figure 8.**
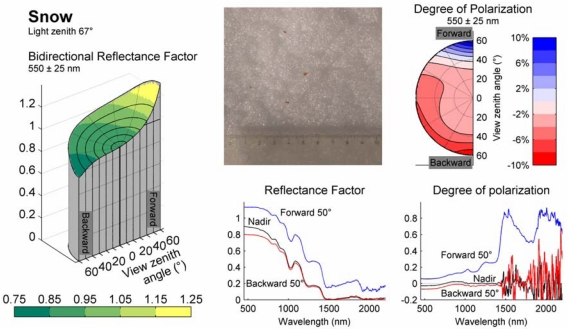
Reflectance properties of a snow sample at 67° illumination zenith angle. (Sodankylä, Finland, April 2008). The sample was measured outside at night time using the laboratory light source. The snow was a few days old and had an icy surface. The subfigures are the same as in Figure 7.

During the last few years, FIGIFIGO has been used in the measurement of more than 150 samples, concentrating on forest understorey vegetation [2,20], snow [21], urban surfaces [22], and airborne sensor reflectance calibration surfaces [23,24]. Polarised measurements have been conducted for approximately 20 samples [25,26]. Two examples from our reflectance library are presented in Figures 7 and 8.

Figure 7 shows a collection of graphs describing reflectance and degree of polarisation of white Spectralon plate as measured with FIGIFIGO in the laboratory. Left-right symmetry is assumed for the Spectralon sample. Degree of polarisation (DOP) is defined as a ratio between difference and sum of horizontally and vertically polarised reflected radiance. White Spectralon proved to have anisotropy in BRF and to polarise weakly, but in relation to other samples it behaves very smoothly. These results are in consensus with equivalent measurements by [27].

Figure 8 shows a similar collection of graphs for a snow sample measured at Sodankylä (Finland) in April 2008. The sample was measured *in-situ* at night time using artificial illumination. The snow was already a few days old and had a hard icy surface. Bulk snow consisted of rounded aggregates a few millimetres in diameter and contained very little liquid water. The icy surface had a strong forward reflectance producing high polarisation in a forward direction.

## 7. Conclusions

The Finnish Geodetic Institute Field Goniospectrometer (FIGIFIGO) is an instrument capable of spectral and polarised bidirectional reflectance factor and hemispherical directional reflectance factor measurements. Most of the design details have been optimised for field operation: the weight, size, and assembly labour are minimised for portability; the system is battery powered to enable measurements anywhere; and a sunphotometer records constantly the incident irradiance to compensate for variations.

In the field, the available time always tends to be limiting the measurement. Thus fast operation has many advantages: (1) a better representativeness can be reached by measuring more samples in a limited time; (2) Measurements are possible and efficient even if measurements must be taken during gaps between clouds; (3) The effects of incident irradiance variation stay small as the Sun position stays more constant during measurement.

In FIGIFIGO, the speeds of transfer and set-up are adequate, but the speed of the actual radiance spectrum collection could still be increased. This could be achieved by improving two features. First, in order to keep FIGIFIGO portable and lightweight, the system is currently lacking an automated azimuth rotation. If a portable lightweight azimuth rail could be added to configuration, without compromising the portability, the operation could be quickened somewhat. Second, the spectrometer of FIGIFIGO can output a spectrum only at ∼600 ms intervals, although sensor integration times are much shorter than this. A faster spectrometer would enable shorter measurement sequences.

Currently, FIGIFIGO reflectances have a general accuracy of 1 – 5% depending on wavelength, sample properties, and measurement conditions. Most of these error bounds are systematic error from calibration, and thus the internal accuracy of data is still much higher; e.g. if an application needs only relative values between two view directions or wavelength bands, the results should have better accuracy.

Although, improvements in radiometry and angle registration are always welcome, these are usually not the key issues in application of field goniospectrometer measurements. The largest challenges are usually related with the bias of sampling. FIGIFIGO is used mostly in measurement of natural samples that vary significantly both spatially and temporally. A single 15-centimeter spot is usually just not large enough to represent the whole species/land cover type, but covers only a strictly defined subset of this. In nature, the species rarely are distributed at clearly separate areas, but land is covered by a mixture of various species. This poses a great challenge for the selection and documentation of representative samples. The only way to decrease the bias is to widen the sampling by repeating the measurements for numerous samples and increasing the optics footprint size.
